# Evaluation of a Self-Guided Eye Movement Training for Individuals with Central Vision Loss

**DOI:** 10.1007/s44402-026-00144-x

**Published:** 2026-07-28

**Authors:** Patricia Grant, Janet P. Szlyk, Meesa Royster, Charlotte C. Jackson, William Seiple

**Affiliations:** 1 The Chicago Lighthouse, Chicago, Illinois USA; 2 Department of Ophthalmology and Visual Sciences, University of Illinois at Chicago, Chicago, Illinois USA; 3 Department of Psychology, Illinois Institute of Technology, Chicago, Illinois USA; 4 Lighthouse Guild, New York, New York USA

**Keywords:** Eye movement training, Macular degeneration, Reading rehabilitation, Vision rehabilitation, Eccentric viewing, Self-guided eye movement training

## Abstract

**Purpose:**

To evaluate reading performance following an 8-week self-guided eye-movement training programme in people with central vision loss.

**Methods:**

Thirty-three adults with a retinal disease affecting the central retina, a documented central scotoma and best-corrected visual acuity between 6/21 and 6/120 in the better-seeing eye were enroled. Of these, 25 participants completed a self-guided eye-movement training programme in a clinical setting, for 2 h per week over 8 weeks. To assess the feasibility of remote delivery, eight participants engaged with the same training platform at home. The primary outcome was maximum reading speed, measured binocularly using Minnesota Low Vision Reading (MNREAD) acuity charts. Visual acuity, depression, adaptation to vision loss and self-reported visual function measures were collected at baseline and following the training period.

**Results:**

Reading speed increased significantly from baseline following training, by an average of 23 + 24.6 words per minute (wpm), representing a 55% gain. Median reading speed increased by an average of 22.6 wpm for the in-clinic group (*n* = 25) and 24.3 wpm for the home-based group (*n* = 8). These findings suggest that self-guided training can enhance reading performance effectively and may be suitable for implementation in both clinical and remote settings.

**Conclusions:**

The self-guided eye movement training programme offers an effective and accessible approach to improve reading performance in individuals with central vision loss. This approach has the potential to complement standard care while broadening access to vision rehabilitation services across both clinical and remote settings.

**ClinicalTrials.gov ID:**

NCT01853930 (5-10-2013)

Key Points

- This study provides evidence that self-guided, structured eye movement training improves reading ability in individuals who have central vision loss.
- The findings suggest that self-guided eye movement training can be delivered as a simple, low-resource intervention, highlighting its potential to enhance the accessibility of vision rehabilitation.
- The results demonstrate the feasibility of implementing self-guided eye movement training in a home setting, with implications for improved access to vision rehabilitation for individuals who cannot regularly attend in-person sessions.

## Introduction

Age-related macular degeneration (AMD) and other retinal diseases that affect central vision remain the leading causes of visual impairment and blindness in the United States, particularly among older adults [1, 2]. As the population continues to age, the prevalence of these diseases is expected to rise, driving greater demand for vision rehabilitation services [3]. Individuals with central vision loss often experience substantial difficulties performing daily activities and detailed tasks independently, such as reading, due to the loss of foveal function [4]. Reduced independence can limit social engagement and have detrimental effects on mental health and well-being, heightening the risk for depression and social isolation [5–9].

Vision rehabilitation programmes aim to help people adapt to central vision loss and maintain their independence. Among the many challenges associated with central vision loss, reading difficulties are a primary concern and a main objective for patients pursuing vision rehabilitation care [10]. Traditional eccentric viewing training is a commonly used vision rehabilitation technique that teaches patients to direct their gaze toward a noncentral preferred retinal locus (PRL), which offers better functioning than the diseased fovea [11–17]. However, even with a well-established PRL, many individuals continue to experience reduced fixation stability, inaccurate saccades and inefficient eye movements that limit reading performance [18–21].

Our 2011 crossover-controlled trial was among the earliest studies to compare different models of reading rehabilitation directly and demonstrated substantial variability in outcomes across approaches [22]. In that trial, reading speeds decreased by an average of 8.4 words per minute (wpm) following Visual Awareness and Eccentric Viewing training, increased by 27.3 wpm after Eye-Movement Training and decreased by 9.8 wpm after Reading Practice with Sequential Presentation of Lexical Information. Rubin et al.’s [20] more recent randomised controlled trial, Eccentric Viewing Training for Age-Related Macular Disease (the EFFECT Study), provides important independent confirmation of this finding, as it also failed to demonstrate a measurable benefit of eccentric viewing training. Together, these findings highlight the inconsistent outcomes associated with eccentric viewing approaches and underscore the need for complementary interventions, such as targeted oculomotor training, that directly address eye movement control and saccadic deficits that eccentric viewing alone may not resolve.

To address oculomotor inefficiencies that accompany PRL use, our previous research focused on developing an eye movement training programme. The programme provides individuals with opportunities to practice controlling and coordinating their eye movements through a series of simple exercises, aiming to enhance eye movement control [22, 23]. Eye movement training focuses on various visual-motor skills beyond eccentric viewing, such as targeted saccades and eye movement control. Outcomes demonstrated consistently that developing eye movement control skills leads to a significant improvement in reading performance, with increases in reading speed by over 46% [22, 23]. These findings underscore the crucial role of effective eye movement strategies in enhancing parafoveal reading abilities.

Building on our previous research, a self-guided version of the eye movement training programme has been developed, specifically designed for independent administration. This includes structured exercises from the original programme, with interactive components that enable users to practice essential eye movement skills independently. By evaluating its impact on reading abilities, the aim is to provide valuable insights into how self-guided training can serve as a viable supplement to traditional rehabilitation strategies. This development comes at a crucial time when an increasing number of individuals require low vision care while the financial demands of clinical resources continue to escalate. Additionally, outcomes were assessed when this eye movement training platform was used remotely. It was hypothesised that all participants would experience similar improvements in their reading skills to those observed with guided training.

## Methods

### Design

This study employed a quasi-experimental design to evaluate reading performance before and after self-guided eye movement control training.

### Participants

Adults diagnosed with a retinal disease affecting the central retina with a documented central scotoma and best-corrected visual acuity ranging between 6/21 and 6/120 in the better-seeing eye were recruited for the study. Persons with other ophthalmologic or neurologic diseases, choroidal neovascularisation, significant media opacities or moderate to severe cognitive impairment were excluded. This study was fully compliant with the Declaration of Helsinki and approved by the Jesse Brown VA Medical Center’s Institutional Review Board. Written informed consent was obtained from all participants.

### Procedures

For each participant, near refraction was measured for each eye at 40 cm and a pair of single-vision near glasses was prescribed and provided for study purposes. Before training, a baseline assessment was conducted that included best-refracted acuity (Early Treatment of Diabetic Retinopathy Study chart), contrast sensitivity (CS) (Mars Letter CS Chart) (Mars Perceptrix; marsperceptrix.com) [24], Patient Health Questionnaire (PHQ-9) [25], Telephone Interview of Cognitive Status (TICS) [26], Adaptation to Age-Related Vision Loss (AVL) scale [27], Veterans Affairs Low-Vision Visual Functioning Questionnaire (VA LV VFQ-48) [28] and reading speed using the Minnesota Low Vision Reading (MNREAD) Acuity Charts (Precision Vision, Inc.; precision-vision.com) [29]. Macular Integrity Assessment (MAIA) fundus-guided microperimetry (Centervue/iCare; icare-world.com) was employed to determine accurately the retinal location of each participant’s centre of fixation. Demographic information was collected, including sex, age, race, employment status, marital status, eye condition and eye disease duration.

### Self-guided Training Platform

The training exercises were run on a 24-inch ASUS All-in-One desktop computer (asus.com). The user interface featured large icons and required only basic computer skills, making it accessible for users with low vision to engage with the programme and exercises. The software enabled control via touch or voice commands and delivered audio instructions for each training activity.

### Eye-movement Training Curriculum

The eye-movement training curriculum includes a series of exercises designed to enhance and refine eye movement skills. Prior work by Duret et al. [30] demonstrated that PRL usage can vary across reading tasks and that the PRL identified during static fixation does not necessarily correspond with the locus employed during dynamic reading. In the present study, participants were not instructed to adopt a specific PRL during training, nor were they instructed on whether they relied consistently on a single locus throughout the intervention period. All training targets were set to a size equivalent to 0.3 log units above the participant’s acuity threshold (smallest identifiable letter size). This size was selected to ensure that the target was visible to participants while still presenting an appropriate level of challenge to promote improvements in eye movement control. Additionally, the super threshold target size reduced sensitivity to slight variations in viewing distance, allowing for more natural and comfortable training sessions. Nevertheless, participants were encouraged to maintain a reading distance of 40 cm. A string indicating the recommended distance was attached securely to the monitor, serving as a visual and tactile reference for participants throughout the training sessions.

Exercises were designed to be easy initially and became more difficult as eye movement skills were gained. Training exercises included locating non-alphabetical stimuli, single letters, letter pairs and words displayed in predictable and random locations on the screen. All training was performed binocularly. The comprehensive training programme and its components are outlined in Table 1 and described in greater detail in previous publications [22, 23].

**Table 1:** Eye movement training exercises.

Training exercises in sequential order		
Exercise name	Description	Subject task
Dot saccade	A dot is alternately displayed at one of two locations on the screen, positioned at 3°, 6° or 9° apart. As training progresses, the rate of alternation (0.5 or 1 Hz) and/or the distance between dots increases.	Saccade between dots as presented.
Letter saccade	A letter is alternately displayed at one of two locations on the screen, positioned at 3°, 6° or 9° apart and changes at randomly selected alternations. As training progresses, the rate of alternation (0.5 or 1 Hz) and/or the distance between letters increases.	Report when the letter changes. Identifying letters is not required
Letter-matching saccade	Pairs of letters are alternately presented at 3°, 6° or 9° apart. Pairs are either identical or different and change at randomly selected alternations.	Report “same” or “different” when pairs change
Word identification saccade	Two- or three-letter words are alternately presented 3°, 6° or 9° apart and change at randomly selected alternations.	Identify words when they change.
Preset search	Starting fixation at a central cross, saccade to single letters presented sequentially at 3°, 6° and 9° from the centre in a clockwise direction.	Identify the letter
Random search	Similar to the Preset Search, but letters appear in random positions.	Identify the letter
Moving window: letter identification	Single letters are presented sequentially across the screen from left to right at distances of 3°, 6° and 9°. With each redraw, the preceding letter disappears and the letter changes at randomly selected intervals.	Identify the letter when it changes.
Moving window: word identification	In separate exercises, two- or three-letter words are presented sequentially from left to right at distances of 3°, 6° or 9°. With each redraw, the preceding word disappears and the word changes at randomly selected intervals.	Identify the word when it changes.
Moving window: sentences	Words that form sentences are presented sequentially across the screen from left to right at distances of 3°, 6° and 9°. With each redraw, the preceding word disappears and a new word appears in the sentence.	Read each word aloud and determine whether the sentence is sensical or nonsensical.

### Orientation

All participants attended an initial in-clinic orientation session where they received training on how to use the training platform. During this initial visit, research staff provided a comprehensive demonstration of the platform’s core features and functionality. They guided the participants through the interface, showing how to navigate and select exercises within the programme, using both touch and voice commands. This hands-on demonstration aimed to enhance participants’ independence and confidence in using the system, ensuring they could customise their training sessions efficiently according to their needs and preferences.

### Clinic-Based Training

Participants in the clinic-based group completed the eye-movement training exercises independently using the platform during scheduled visits. Participants were required to engage in one 2-h training session per week for a total of 8 consecutive weeks. During each session, research staff provided encouragement and real-time feedback. This self-guided approach aimed at maximising training effectiveness while fostering confidence in using the platform autonomously.

### Home-Based Training

To explore using the training platform remotely, a subset of participants used the training platform in their home setting. Assignment to the home-based group was based on preference. Individuals who expressed a preference for completing the training at home and who had adequate space to train and to follow the home-based protocol were assigned to the home-based condition. After the orientation session, a member of the research team delivered and set up the training platform in each participant’s home. Participants were encouraged to train as often as they desired but were required to complete a minimum of 2 h of training per week for 8 consecutive weeks. Weekly phone calls were conducted to offer encouragement, monitor progress and address any questions or technical issues. Participants were also provided with a helpdesk phone number to report any technical issues. This approach fostered participant independence while maintaining ongoing support and motivation throughout the training period.

### Primary Outcome Measure

Binocular reading performance was measured at two key time points: at baseline before the start of the training and again after completion of the 8-week programme. The assessments utilised printed MNREAD charts, which consist of sentences of decreasing font size designed to evaluate reading acuity and maximum reading speed (MRS), measured in wpm. Participants were instructed to read each sentence aloud as accurately and quickly as possible. Reading speed for each sentence was calculated from the recorded reading time of words read correctly, and these measurements were then used to generate a reading-speed curve. MRS was derived from the plateau region on the curve, at larger print sizes, where reading performance was not limited by print size. This approach follows the original MNRead scoring method [29]. To minimise the learning effect, two versions of the charts were used. All assessments were administered by the same examiner across participants and sessions to reduce measurement variability.

### Data Analysis

Data were summarised with descriptive statistics. Pearson’s correlation tests were conducted to evaluate the relationship between baseline vision measures and cognitive and psychological assessments and reading performance after training. Paired *t*-tests and the Wilcoxon signed-rank test were employed to assess changes in baseline measures following training using IBM SPSS v. 20 software (ibm.com)

The VA LV VFQ-48 was analysed using Rasch measurement theory with Winsteps v. 4.8.2 (winsteps.com). Person measures were calculated for each participant using the standard Rasch scoring file, which applies the fixed item difficulty calibrations published by Stelmack et al. [31]. This approach provided precise estimates of participant abilities across each of the questionnaire’s five domains, with higher person measures indicating greater ability [32]. Subsequently, paired *t*-tests were conducted to compare the person measure for each domain (overall visual ability, reading, mobility, visual information processing and visual motor skills) before and after training, assessing any statistically significant changes resulting from the intervention. Lastly, a multivariate linear regression analysis was conducted to identify potential predictors of change in reading speed following training.

## Results

### Baseline

Thirty-three participants (21 women, 12 men), ranging in age from 25 to 97 years (mean age = 68 years), were enroled in the study. Sixty percent of participants had a diagnosis of AMD and 21% had Stargardt macular dystrophy. Sixty-one percent of the participants were white, 27% black and 9% Asian. Forty percent were employed, 15% unemployed and 45% retired. The average self-reported time since diagnosis was 9.9 years, with a range spanning from 2 to 32 years. The mean binocular visual acuity at baseline was 0.95 ± 0.24 log MAR or 6/54 Snellen equivalent, mean CS was 1.03 ± 0.42 log CS (normal range 1.72–1.92) and mean binocular baseline MRS was 55.7 + 40.2 wpm. Fixation point eccentricity ranged from 0.1°, indicating closer proximity to the fovea, to 10.6°.

### Participant Characteristics by Group

In the home-training group, there were five women and three men, with a mean age of 75.9 ± 11.9 years. The mean duration of vision loss was 14.3 ± 10.1 years, mean visual acuity was 1.0 ± 0.3 logMAR, CS was 1.1 ± 0.4 logCS and baseline reading speed averaged 40.9 ± 35.6 wpm.

In the clinic-based group, there were 16 women and nine men, with a mean age of 65.6 ± 24.1 years. The mean duration of vision loss was 8.6 ± 5.9 years, mean visual acuity was 0.90 ± 0.20 logMAR, CS was 1.0 ± 0.4 logCS and baseline reading speed averaged 60.4 ± 41.1 wpm. There were no significant differences between the two groups at baseline in terms of age, race, visual acuity, CS, TICS, PHQ-9, AVL scale scores and reading speed.

Detailed individual characteristics are summarised in Table 2.

**Table 2:** Baseline demographic and clinical characteristics of participants.

ID	Group	Age (years)	Sex	Duration (years)	Diagnosis	Visual acuity (logMAR)	Contrast sensitivity (logCS)	Baseline reading speed (wpm)
1	Clinic	25	F	4	Stargardt	0.60	1.64	115.2
2	Clinic	26	F	13	Stargardt	0.70	1.48	111.7
3	Clinic	34	F	5	Stargardt	0.60	1.88	117.9
4	Clinic	47	M	10	Stargardt	0.70	1.64	98.5
5	Clinic	51	F	12	Cone-Rod Dystrophy	0.90	1.04	43.0
6	Clinic	51	M	5	PDR	1.30	0.84	17.8
7	Clinic	94	F	10	AMD	1.10	0.56	34.3
8	Clinic	83	F	10	AMD	1.24	0.96	16.2
9	Clinic	77	M	2.5	AMD	1.00	0.52	38.4
10	Clinic	62	M	7	AMD	0.70	0.68	29.6
11	Clinic	29	M	2	Stargardt	0.90	1.32	106.7
12	Home	83	F	15	AMD	0.90	1.36	74.5
13	Clinic	97	F	5	AMD (Dry)	1.00	0.88	67.8
14	Home	75	F	26	Myopic Degeneration	1.30	0.64	40.2
15	Clinic	94	F	5	AMD	1.00	0.96	28.3
16	Home	55	M	6	Macular Dystrophy	0.70	1.44	111.8
17	Home	63	F	32	Stargardt	1.20	1.28	17.5
18	Clinic	82	F	5	AMD	1.00	1.20	56.9
19	Clinic	76	F	5	AMD	0.90	1.36	132.7
20	Clinic	25	M	6	Stargardt	0.90	1.12	75.1
21	Clinic	85	F	10	AMD	1.30	0.36	10.0
22	Home	92	F	4	AMD Dry	1.30	0.28	8.5
23	Clinic	84	F	7	AMD	0.60	1.24	87.4
24	Home	84	F	10	AMD	0.90	1.08	13.5
25	Clinic	73	F	8	AMD	0.90	0.64	38.8
26	Clinic	51	M	31	RP	1.20	0.52	20.5
27	Home	80	M	6	AMD	0.60	1.20	22.0
28	Clinic	56	M	15	DR	1.00	0.68	10.2
29	Clinic	88	F	15	AMD	1.20	0.92	101.8
30	Home	75	M	15	AMD	0.90	1.32	39.3
31	Clinic	87	F	5	AMD Dry	1.20	0.40	32.2
32	Clinic	81	F	7	AMD	0.60	1.64	108.8
33	Clinic	83	M	10	AMD	1.20	0.88	10.1

*AMD* age-related macular degeneration, *DR* diabetic retinopathy. *F* female, *M* male, *wpm* words per minute, *PDR* proliferative diabetic retinopathy, *RP* retinitis pigmentosa.

Mean baseline scores on the TICS were 33.2 ± 2.5, with no participants falling in the mild to moderate cognitive impairment range. Scores on the PHQ-9 averaged 3.1 ± 4.5. Twenty-six participants had scores consistent with the absence of depression, four had scores reflecting mild depressive symptoms, one was classified as experiencing moderate depression and two exhibited scores indicative of severe depression. Scores on the AVL scale averaged 55.1 ± 10.8, ranging from 25 to 69. Higher scores on the AVL scale reflect better adaptation to vision loss.

Age, visual acuity and CS at baseline were not significantly correlated with the TICS, PHQ-9 or AVL scale baseline measures. A strong inverse correlation was observed between the AVL scale and the PHQ-9 scores (*r *= −0.71, *p* < 0.001), indicating that greater difficulty in adapting to vision loss was associated with higher levels of depressive symptoms. No significant associations were found between diagnosis, TICS, PHQ-9 or AVL scale and the changes in reading speed following training, suggesting that improvements in reading performance were independent of disease category, baseline measures of cognitive status, depressive symptoms or adaptation to vision loss. At baseline, significant correlations were observed among age, visual acuity, CS and reading speed (all *p*-values < 0.01; Table 3).

**Table 3:** Pearson correlation coefficients (*r*) among age, visual acuity, contrast sensitivity and reading speed at baseline.

	Baseline measures		
Baseline measures	Visual acuity (logMAR)	Contrast sensitivity	Reading speed (words per minute)
Age	0.62	−0.74	−0.43
Visual acuity		−0.73	−0.64
Contrast sensitivity			0.74

All correlations were significant (*p* < 0.01).

### Pre-Post Training Results for All Participants (*n* = 33)

Following eye movement training, all participants demonstrated significant improvements in both visual acuity and CS (paired *t*-tests: *p* = 0.01 and *p* < 0.001, respectively). In contrast, scores on the PHQ-9 and AVL scale did not show significant change post-training (Wilcoxon signed-rank test: *p* = 0.46 and 0.22, respectively). Notably, all five domains on the VA LV VFQ-48 showed statistically significant improvement, indicating enhanced self-reported visual functioning across multiple domains (Table 4).

**Table 4:** Pre- and post-training person measures (logits) across domains of the Veterans Affairs Low Vision Visual Functioning Questionnaire-48 (VA LV VFQ-48), showing means, ranges and paired *t*-test results; higher scores indicate better perceived functioning.

Domain	Pre-training		Post-training			
	Mean (SD)	Range	Mean (SD)	Range	*t*-value	*p*-value
Overall visual ability	1.36 (0.97)	−0.10 to 3.14	1.87 (0.72)	0.61–3.26	−4.13	<0.001
Reading	1.62 (1.87)	−1.57 to 6.76	2.26 (1.28)	−1.57 to 5.12	−2.51	0.02
Mobility	1.31 (0.98)	−0.59 to 3.01	1.85 (0.75)	0.57–3.24	−4.10	<0.001
Visual information	1.47 (0.99)	−0.16 to 3.04	1.85 (0.76)	0.24–3.56	−3.14	0.004
Visual motor	1.14 (0.87)	−0.53 to 2.65	1.55 (0.71)	−0.25 to 2.65	−3.60	0.001

**Fig. 1:**
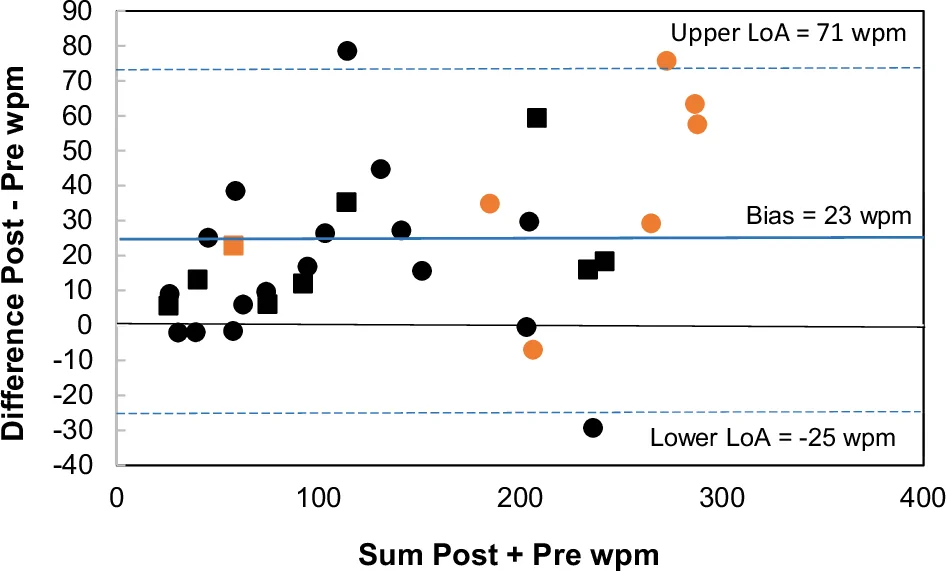
Bland–Altman plot illustrating the relationship between combined reading speeds (sum of pre- and post-training values on the *x*-axis) and the change in reading speed (post- minus pre-training on the *y*-axis). The mean difference (bias) was 23 words per minute (wpm), with limits of agreement (LoA) ranging from −25.2 to 71.2 wpm. The 95% confidence intervals were −39.8 to −10.7 (lower) and 56.7 to 85.8 (upper). Reflecting variability in individual responses, 27 participants improved, five showed minimal or no change and one experienced a decline in performance. Participants with Stargardt disease are shown as orange data points and those with age-related disease are shown in black. Clinic and home-based data points are shown as circles and squares, respectively.

To identify patterns or potential outliers in the data, Fig. 1 presents a Bland–Altman plot [33] illustrating the relationship between combined reading speeds (the sum of pre- and post-training values) and the change in reading speed (post- minus pre-training) across all participants. On average, reading speed improved significantly following training, with a mean increase of 23 ±  24.6 wpm, representing a 55% gain over baseline across participants. Bland–Altman analysis demonstrated a mean difference (bias) of 23 wpm, with limits of agreement ranging from –25 to +71 wpm, reflecting considerable variability in response. Consistent with this observed variability in individual responses, 27 participants improved, five showed minimal or no change and one exhibited a decline in reading performance.

The outlier was a 76-year-old woman diagnosed with AMD, who, unlike the majority of the participants, experienced a decline in reading speed following training. Her reading speed decreased by 29 wpm, from 132.7 at baseline, one of the highest in the group, to 103.7 wpm post-training. This reduction exceeded two standard deviations below the mean change observed across all participants, marking a statistically and clinically significant deviation. Her baseline PHQ-9 score was two, indicating minimal depressive symptoms and her AVL scale score was 67, reflecting relatively high adaptation to vision loss. Visual acuity at baseline was 6/37.5 Snellen and CS measured 1.3. Notably, during the study, her fixation eccentricity increased by 4° and her fixation stability, quantified by the Bivariate Contour Ellipse Area using the MAIA microperimeter, increased by a factor of two, suggesting a marked shift in oculomotor behaviour that may have contributed to her reduced reading performance.

Lastly, the multivariate linear regression analysis did not identify any baseline demographic or clinical variables, such as age, visual acuity, CS or other factors to be significant predictors of change in reading speed following training.

### Evaluation by Training Setting

**Fig. 2:**
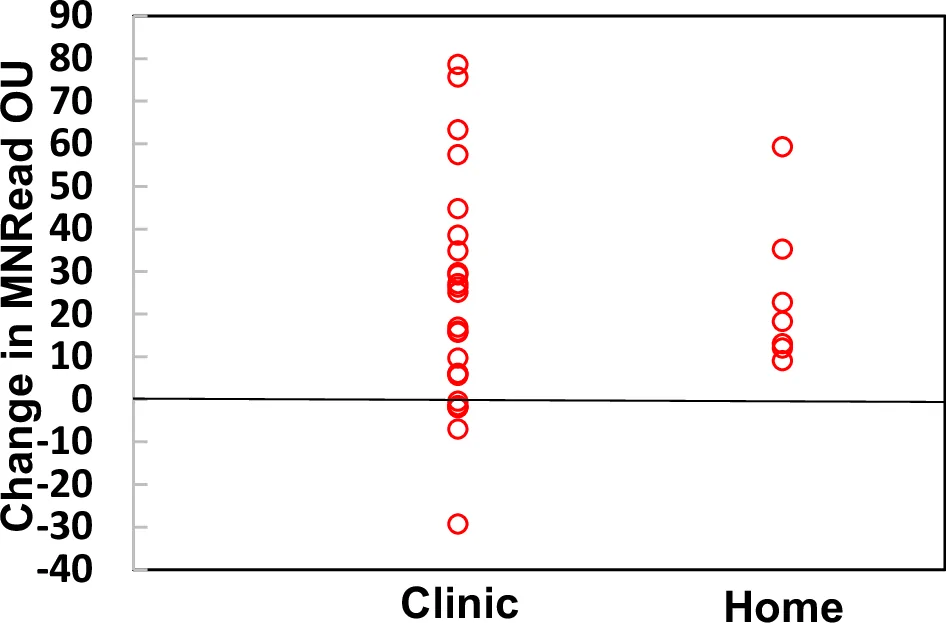
The graph displays increases in words per minute (wpm), with participants in the home-based training group showing a mean increase of 24.3 wpm and the clinic-based group improving by 22.6 wpm. MNRead OU, Minnesota Low Vision Reading Acuity Chart read binocularly.

Figure 2 illustrates that gains in reading speed achieved through home-based training (mean increase: 24.3 wpm) were comparable to those observed in the clinic setting (mean increase: 22.6 wpm). These findings highlight the potential viability of remote interventions and underscore the need for a rigorously designed study to compare the efficacy of home-based versus in-clinic training approaches directly.

## Discussion

The importance of comprehensive vision rehabilitation is well-established in the literature, recognising its vital role in improving the quality of life for individuals with visual impairments. Among the core objectives of vision rehabilitation, improving reading performance stands out as a key priority. The ability to read, whether print or digital content, is fundamental to daily functioning, enabling individuals to work, learn, access information, communicate and engage in leisure activities. Rehabilitation strategies that specifically address reading challenges can play a transformative role in restoring functional independence, fostering autonomy and promoting overall well-being.

The gains observed in the present study, 22.6 wpm for the in-clinic group and 24.3 wpm for the home-based group, represent substantial improvements in functional reading performance. To place these changes in context, it is important to consider the degree of variability expected in the absence of training. In our 2011 study, where a randomised, repeated-measures, counterbalanced crossover design was employed to evaluate three forms of reading rehabilitation, the no-intervention control group remained essentially unchanged in reading performance after 18 weeks (0.96 ± 1.3 wpm) [22]. This minimal fluctuation reflects the expected test–retest variability in reading speed for individuals with central vision loss.

Similar findings regarding test–retest performance have been reported in other studies using MNRead Charts, which have shown that MRS is a relatively stable and repeatable outcome measure in individuals with macular disease. In particular, Patel et al. [34] and Subramanian and Pardhan [35] reported low test–retest variability for MRS in individuals with AMD, and a review by Brussee et al. [36] concluded that MRS demonstrates good repeatability compared with other reading metrics. Together, these findings suggest that the improvements observed in the present study far exceeded expected measurement variability and are therefore likely to represent true training-related gains in reading performance rather than measurement variability. The findings of this study demonstrate that a self-guided training programme can improve reading performance effectively in individuals with central vision loss, offering a promising and scalable solution for enhancing functional outcomes.

Importantly, the results suggest that training conducted outside of a clinical setting may improve reading performance, underscoring the potential for remote rehabilitation approaches. These outcomes contribute to a growing, but still limited body of literature examining the efficacy of home-based reading interventions. Ngyuen et al. [37] implemented a 4-week at-home training protocol with 36 participants, focused on rapid serial visual presentation (RSVP) reading with optimal visual aids and reported a 25% improvement in reading speed for small print (10 pt font). Coco-Martin et al. [38] employed a hybrid model combining in-clinic and at-home practice using prescribed low-vision aids, yielding substantial gains in printed text reading; findings consistent with Frennesson et al. [39] and Nilsson [40]. Silvestri et al. [41] conducted 14 consecutive training sessions at home, each lasting 45 min, and observed a 33% increase in reading speed on the MNRead charts. Collectively, these studies reinforce the feasibility and effectiveness of home-based interventions for improving reading function in individuals with visual impairments.

### Limitations

Although the current study did not include a no-training control group, previous research has demonstrated that eye movement training leads to significant improvements in reading performance when compared with control conditions [22].

## Conclusions

As the population ages and the prevalence of eye diseases rises, innovative solutions are needed urgently to meet the growing demand for vision rehabilitation services. The self-guided eye movement training programme, which uses simple, low-cost equipment, adaptable to both clinical and home environments, offers a practical and scalable approach to enhancing standard care. Its flexibility allows for personalised pacing and repeated practices, fostering sustained improvements in reading speed and overall functional independence. Low vision practitioners can incorporate this programme into routine care as a supplemental tool for independent training, helping patients to maintain or improve reading performance outside of traditional clinical settings. By bridging gaps in service delivery, this model promotes equitable access to rehabilitation support across diverse populations and geographic regions.

## Data Availability

No datasets were generated or analysed during the current study.
